# Tutorial on using EEG microstates to study moment-to-moment large-scale functional brain network dynamics in neurodevelopment

**DOI:** 10.3389/fnins.2026.1891985

**Published:** 2026-09-14

**Authors:** Priyanka Ghosh, Ana C. Sobrino, Kirsten A. Donald, Laurel J. Gabard-Durnam

**Affiliations:** 1 Institute for Cognitive and Brain Health, Northeastern University, Boston, MA, United States; 2 Department of Psychology, Northeastern University, Boston, MA, United States; 3 Division of Developmental Paediatrics, Department of Paediatrics and Child Health, Red Cross War Memorial Children’s Hospital, University of Cape Town, Cape Town, South Africa; 4 The Neuroscience Institute, University of Cape Town, Cape Town, South Africa

**Keywords:** developmental populations, EEG microstate analysis, HAPPE, large scale brain networks, neurodevelopment, tutorial

## Abstract

EEG microstates are topographical patterns of EEG activity that offer a lens into the fast temporal dynamics of large-scale functional network activity and whole-brain functional organization. Microstates can capture temporal patterns of network configurations and transitions between these configurations at the millisecond scale. Although extensively studied in adults, microstates remain underutilized in developmental neuroscience, despite their potential for tracking significant developmental changes in brain network dynamics indicated by developmental fMRI studies. Extracting EEG microstates from developmental populations with relatively high artifact levels compared to adults remains a challenge due to the sensitivity of the method to such artifacts. Our tutorial provides a practical guide that walks the readers through the microstate analysis pipeline with a focus on special considerations for developmental data within the free, open-source software HAPPE (Harvard Automated Processing Pipeline for Electroencephalography) that is optimized for developmental artifacts. We first detail the conceptual foundations and current status of EEG microstates in developmental literature and then present a step-by-step tutorial on computing and interpreting microstates from resting-state and task-based EEG in developmental cohorts. All de-identified datasets used in the tutorial and the annotated microstate analysis code are freely available for public access at https://zenodo.org/records/17527065 and https://github.com/PINE-Lab/HAPPE/blob/master/3.%20generate/generateMicroStates.m. This article aims to make microstate computation more accessible, robust, and reproducible in developmental cognitive neuroscience.

## 1 Background

EEG microstates are brief, recurrent topographical patterns of scalp EEG activity that remain stable for approximately 60–120 ms before rapidly transitioning to another configuration (Lehmann et al., 1987, 1998). Microstates reflect patterns of coordinated neural activity that dominate the dynamics of large-scale functional network activity and whole-brain organization from moment-to-moment (Khanna et al., 2015; Michel and Koenig, 2018). Their millisecond-scale temporal resolution allows researchers to study transitions between global network configurations in real time, offering a powerful complement to fMRI measures of large-scale connectivity (Britz et al., 2010). Unlike fMRI, which captures slow (in the order of seconds) hemodynamic coupling among distributed brain regions, EEG microstates provide millisecond-resolved access to the rapid reconfiguration of global neural activity patterns that underlie these networks. Interestingly, it has been shown that the large-scale functional networks observed with fMRI reflect the long-time averages of these rapidly switching metastable patterns (Baker et al., 2014; Deco et al., 2011; Preti et al., 2017; Vidaurre et al., 2017). Microstates can reveal the rapid occurrence, sequence, and duration of the network activation states that are temporally blurred in fMRI estimates, providing insights into network flexibility, network stability, and neural efficiency (Britz et al., 2010; Khanna et al., 2015; Michel and Koenig, 2018).

Developmental neuroimaging studies indicate extensive and significant organization in the functional as well as the structural architecture of the brain from neonatal ages onwards (Fransson et al., 2011; Gao et al., 2015; Smyser et al., 2016). However, microstates remain underutilized in developmental neuroscience to understand the changes in large-scale functional networks. To facilitate developmental research and address an important gap in understanding large-scale brain activity development using microstates, we provide a tutorial that (a) reviews important microstate concepts from existing literature and (b) provide a detailed step-by-step tutorial on computing and interpreting microstates implemented within the MATLAB-based (The Mathworks, Inc.) HAPPE software (Gabard-Durnam et al., 2018; Lopez et al., 2022; Monachino et al., 2022; Xu et al., 2024) using example datasets from 6-month-old and 2-year-old children. These datasets are freely available for public access to reproduce tutorial analyses athttps://zenodo.org/records/17527065. Together, this tutorial aims to facilitate a more streamlined, transparent, and user-informed approach to EEG microstate computation and interpretation in developmental cognitive neuroscience.

## 2 EEG microstates: foundational concepts

EEG microstates reflecting different neural activity patterns can be computed from both resting-state and task-based EEG recordings (Nazare and Tomescu, 2024; Seitzman et al., 2017). Once computed, several statistical features can be extracted to characterize their temporal dynamics and sequence. The *Global Explained Variance (GEV)* represents the total explained variance in the data, explained by a given microstate class. The *duration* of a microstate represents the average time a given topographical class remains stable before transitioning to another configuration, reflecting the temporal dominance of a specific functional network. The *occurrence* quantifies how frequently a microstate class appears per second, indexing how often the brain re-engages that functional configuration. The *coverage* refers to the proportion of total recording time occupied by each microstate class, providing a measure of its relative dominance within the overall brain activity. Finally, *transition probabilities* capture the likelihood of switching from one microstate class to another, representing the ordered succession of microstate occurrences over time. All these measures provide a window into the brain’s moment-to-moment functional organization and can be readily computed with the generateMicroStates.m functionality within HAPPE to compare the properties of network dynamics across developmental stages or experimental conditions.

Specific EEG microstate classes have been repeatedly linked to distinct large-scale neural networks and cognitive functions, although some inconsistencies exist in adult literature regarding their classification and functional interpretation. Canonically, four microstates, labeled with letters A-D, are observed during resting-state EEG in adults (Khanna et al., 2015; Michel and Koenig, 2018; Tarailis et al., 2024). Microstate A has been primarily related to sensory (auditory and visual) processing and general arousal, whereas microstate B appears more closely tied to visual and visuospatial networks and, in some studies, to self-visualization or imagery processes (Michel and Koenig, 2018; Milz et al., 2016; Seitzman et al., 2017). Microstate C is linked to the default-mode network (DMN) and internally directed processes and typically shows reduced occurrence during tasks requiring externally oriented attention (Custo et al., 2017; Milz et al., 2016). In contrast, microstate D has been associated with the dorsal attention and executive control networks; its duration increases significantly during attentionally demanding tasks, reflecting heightened engagement of attentional control mechanisms (Britz et al., 2010; Khanna et al., 2015). Recent research has systematically quantified additional microstate classes (Custo et al., 2017; Koenig et al., 2024) where microstates E, F and G have been related to the salience, anterior default mode network, and sensorimotor networks, respectively (see review by Tarailis et al., 2024 for more details). These findings suggest that different microstate classes capture transient neural states that differentially engage sensory, associative, and attentional networks at rest. It is important to note here that all functional associations reviewed here involved participants from the adult population.

When computed during tasks, EEG microstates index the moment-to-moment engagement, disengagement, and reconfiguration of large-scale neural networks supporting perception, attention, affect, and cognition (Han et al., 2025; Michel et al., 2024). For example, using a mental arithmetic task, Kim et al. (2021) reported how attention-related microstate D is positively associated with cognitively demanding trials, while DMN-related microstate C is associated negatively with those same demanding trials. In socio-affective contexts, microstates have been shown to represent discrete emotional states, and their temporal fluctuations enable decoding of emotion dynamics across conditions (Schiller et al., 2023; Zerna et al., 2021). The temporal dynamics of microstates can also distinguish between cognitive components of fluid reasoning as well as mediate the influence of past outcomes on decision-making (Pedroni et al., 2016; Zappasodi et al., 2019). Collectively, task microstate properties can discriminate between cognitive functions, scale with cognitive demands, track affective dynamics, and predict individual differences in task performance.

## 3 Development of EEG microstates

EEG is a non-invasive, affordable, and scalable tool that can be applied readily and longitudinally from postnatal ages onwards. In pediatric populations, where MRI or MEG recordings from awake, behaving participants may be challenging or poorly tolerated, EEG microstates can capture the dynamics of spontaneous and task-related large-scale brain networks (Britz et al., 2010; Ghosh et al., 2025; Koenig et al., 2002). Moreover, microstate dynamics can provide important insights about both neurotypical and neurodivergent developmental paths in large-scale brain function (Takarae et al., 2022; Tomescu et al., 2015) with potential translational value for early identification and support efforts (see review by Das et al., 2022).

Early work by Koenig et al. (2002) demonstrated that EEG microstate maps and parameters evolve systematically from childhood to adulthood. In early-to-middle childhood (4–12 years), for example, both duration and occurrence for microstate E were found to be positively associated with age for the eyes-open condition but not the eyes-closed resting state (Takarae et al., 2022). In children aged 4–8 years, task-related EEG microstate measures during a go/no-go task were found to be associated with error-related negativity (ERN) responses, providing evidence for developmentally relevant neural signatures of cognitive control (Bagdasarov et al., 2024b). Developmental studies have shown increased microstate duration and decreased occurrence in development compared to adults, possibly reflecting immature or unstable network states at an early age (Koenig et al., 2002). Such developmental trends parallel known trajectories in white matter myelination, synaptic pruning, and network specialization observed through MRI-based measures (Adibpour et al., 2017; Fair et al., 2009; Lippé et al., 2007; Supekar et al., 2009).

Recent studies suggest that EEG microstates provide a powerful window into the rapidly evolving architecture of large-scale brain network reorganization across early development. Brown and Gartstein (2023) reported systematic age-related changes in microstate properties across 6–10 months in infancy, confirming developmental sensitivity within the first year. In another study by Adibpour et al. (2025), pre-term infants showed slower microstate dynamics (i.e., longer durations and fewer occurrences) than full-terms at term-equivalent age, indicating less mature brain function, more in males than females. During naturalistic caregiver–infant play, microstate D duration associated positively with infant-led joint attention and sustained attention, suggesting an association between the maturation and stability of microstate D with developing attentional control abilities (Bagdasarov et al., 2025). Similarly, microstate D in response to faces with direct gaze was the most consistently informative of categorical ASD outcome and dimensional variation in social skills (Gui et al., 2021). In the largest infant study to date, Ghosh et al. (2025) examined 854 infant EEG recordings longitudinally across multiple cohorts to demonstrate more rapid microstate transitions with development, particularly during early infancy. This longitudinal study also provided data-driven evidence of functional network specialization in growing infants, with microstates corresponding to sensorimotor networks emerging in the second year of infancy. As indicated by emerging neonatal and preterm infant studies, microstate features track development from very early life, but most datasets to date are small and methods heterogeneous, making cross-study comparisons and consistencies difficult to parse (Hermans et al., 2024; Khazaei et al., 2021). Harmonized methods and measures to capture early brain network dynamics across developmental populations would facilitate understanding these critical processes (Zanesco et al., 2020).

## 4 EEG microstates in clinical neurodevelopment

EEG microstates can provide early insights into functional alterations in clinical populations by capturing disorder-specific differences in large-scale neural networks in a more accessible and cost-effective manner compared to other neuroimaging modalities. Autistic children show altered brain network dynamics as indexed by EEG microstates compared to typically developing groups in recent studies (Bochet et al., 2021; Gui et al., 2021; Kang et al., 2026; Wei et al., 2025). For example, Nagabhushan Kalburgi et al. (2020) reported that in children with autism spectrum disorder (ASD), the duration of microstate C was increased and its frequency decreased compared to their typically developing (TD) peers in eyes-closed resting state. In their study, Takarae et al. (2022) reported that the mean duration of microstate C was negatively correlated with ADOS Social Communication scores that reflect an individual’s severity of social and communicative behaviors. Functionally, these results support explanations for the difficulty individuals with ASD have in processing environmental social cues. Altered default-mode associated microstate dynamics have also been reported in children and adolescents with Avoidant/Restrictive Food Intake Disorder (Bhadra et al., 2025) and in adolescents diagnosed with major depressive disorder (MDD) (Hu et al., 2023; Zhang et al., 2025). This emerging pattern of results suggests Microstate C in particular may be sensitive to changes in a wide range of clinical conditions.

Altered sensory-related microstates have also been observed in clinical neurodevelopment, though without consistency across conditions in the available limited studies. Studies in pediatric attention-deficit/hyperactivity disorder (ADHD) show subtype-specific alterations in sensory microstate parameters as compared to healthy controls. Specifically, microstate B was found to differentiate children with the ADHD-combined type (ADHD-C) from those with the inattentive subtype (ADHD-I) (Berchio et al., 2025; Luo et al., 2021, 2022). Adolescents diagnosed with MDD have shown increased occurrence of microstate A and decreased transition probability of microstate A→C as compared to healthy adolescents, suggesting altered sensory and sensory-default mode network dynamics (Hu et al., 2023; Zhang et al., 2025). Overall, these findings support microstate analysis as a non-invasive, millisecond-resolved clinically relevant marker of large-scale neural network differences sensitive to symptom levels, treatment state, and clinical progression across developmental populations.

## 5 Current microstate methodology

Multiple software and libraries exist for computing microstates, including EEGLAB-based toolboxes such as the Microstate EEGlab Toolbox (Poulsen et al., 2018) and Microstatelab (Nagabhushan Kalburgi et al., 2024), Windows-based Cartool (Brunet et al., 2011), the MATLAB toolbox +microstate for sensor/source-space analysis (Tait and Zhang, 2022), MNE-Python based pycrostates (Férat et al., 2022), and Brainstorm’s CENA microstates (Cacioppo and Cacioppo, 2015). Across platforms, visual inspection of maps for neurophysiological plausibility (e.g., bilaterality, anterior–posterior gradients) remains critical, especially in pediatric datasets with low signal-to-noise ratio. Most of these toolboxes standardize the microstate computation workflow, but it remains crucial to report preprocessing and clustering choices, to justify the number of maps with objective criteria, and to interpret map-behavior links probabilistically in developmental populations where normative patterns still need to be established.

EEG microstate analysis typically begins with the identification of periods of stable scalp topographies in the EEG dataset, segmented at local peaks of Global Field Power (GFP) representing time points of maximal global neural synchrony (Lehmann et al., 1987; Michel and Koenig, 2018). Most toolboxes rely on a polarity-invariant clustering algorithm, such as modified k-means or atomize and agglomerate hierarchical clustering (AAHC), to derive prototypical topographic maps (microstate classes), which are computed by grouping similar scalp voltage topographies (Poulsen et al., 2018). To ensure that only the most representative and stable patterns are used, topographies are typically extracted at the GFP peaks, which mark moments of strongest and most reliable global neural activations. Clustering is unsupervised and aims to minimize the spatial dissimilarity (Global Map Dissimilarity). After clustering, each time point in the individual datasets is assigned a label of its best-matching class (backfitting), and temporal features such as duration, occurrence, coverage, and transition probabilities are extracted per class. Toolboxes like Microstatelab (Nagabhushan Kalburgi et al., 2024) and Cartool (Brunet et al., 2011) use a two-step approach to determine the prototypical topographic maps, where clustering is first performed at the individual level to derive participant-specific template maps, followed by submitting them to a second clustering step to derive group-level prototype maps. The Microstatelab toolbox (Nagabhushan Kalburgi et al., 2024) also offers an additional test to statistically identify the topographical differences between groups and conditions, which makes it superior. The Microstate EEGlab toolbox (Poulsen et al., 2018) on the other hand uses a single-step group-level prototype map derivation approach, followed by estimating goodness-of-fit measures like Global Explained Variance (GEV) and cross-validation criterion (CV) to determine the optimal number of microstate classes. The data-driven cross-validation approach helps control for clustering such that the solution reflects actual neural activity instead of unrelated recording noise. This is particularly helpful when dealing with noisy developmental datasets where normative microstate patterns still need to be established.

## 6 Current limitations

Despite the growing enthusiasm for EEG microstate analysis as a tool for indexing large-scale brain dynamics, several methodological inconsistencies constrain its interpretability and reproducibility. A key challenge lies in the lack of standardization across preprocessing and clustering pipelines, including differences in filtering parameters, reference schemes, artifact correction methods, and reliability criteria for stable microstate maps (Michel and Koenig, 2018; Milz et al., 2017). Such variability can yield divergent microstate maps and temporal parameters, complicating cross-study comparisons (Haydock et al., 2025). The choice of the number of microstate classes, whether restricted to the canonical four (A-D) or extended classes (E-G) remains somewhat arbitrary, often determined by heuristic or data-dependent measures (Custo et al., 2017). Associations between microstate maps and their corresponding functional networks are probabilistic as they are inferred from EEG-fMRI correlations and source models (Britz et al., 2010). They can vary across task conditions and ages, making the physiological interpretation of one-map/one-network labels difficult (Tarailis et al., 2024). Additionally, while microstates are hypothesized to reflect transient activations of large-scale functional networks, direct links to neural sources remain incompletely understood in infants and children, where source reconstruction is challenging because volume conduction properties may not apply as in adults due to smaller head sizes, thinner skulls, and different folding patterns in the cortices.

Another major limitation is the lack of large, normative microstate datasets across development, which hinders robust modeling of inter-individual variability (though see Ghosh et al., 2025). There are also scarce longitudinal studies to understand within-individual changes in microstate dynamics during development. Tutorials by Bagdasarov et al. (2024a) have demonstrated the feasibility and reliability of microstate computations in development using Cartool (Brunet et al., 2011), a GUI-based software that runs only on the Windows operating system. However, the absence of accessible scripts within a commonly used MATLAB/EEGLAB framework makes batch processing and large-scale automation of microstate analyses difficult. Movement, variable vigilance, and shorter recordings are all common in developmental EEG, which is often noisy (Gabard-Durnam et al., 2018; Monachino et al., 2022). These factors may obscure noisy or invalid segmentation for estimating microstates if not handled with age-appropriate pipelines and quality control measures. Addressing these methodological challenges through open-source, standardized, and developmentally sensitive pipelines such as those outlined in this tutorial will be critical for advancing the interpretability and translational impact of microstate research in developmental populations.

## 7 A step-by-step tutorial for computing microstates in HAPPE

EEG microstate analyses can be sensitive to the preprocessing steps used to clean developmental EEG data due to elevated artifact prevalence and shorter recording durations. To mitigate these challenges, researchers can preprocess their EEG data using HAPPE, which now provides a freely accessible integrated script *generateMicroStates.m* (Github link) that wraps around the Microstate EEGLAB Toolbox (Poulsen et al., 2018). The *generateMicroStates.m* functionality computes microstates using developmentally sensitive parameters and automates all microstate analysis steps from preprocessed EEG data loading → GFP peak detection → segmentation → backfitting → smoothing → metric extraction and export to.csv file format.

For researchers new to HAPPE, here is an outline of the software requirements and setup needed to preprocess developmental EEG data and subsequently perform microstate analysis.

i. *Software versions and operating systems*: HAPPE version 4 or later with MATLAB R2022b or later (tested on both macOS and Windows 11).
ii. *MATLAB toolbox requirements*: Signal Processing Toolbox, Optimization Toolbox, Statistics and Machine Learning Toolbox, and Wavelet Toolbox.
iii. *Downloading HAPPE*: HAPPE can be downloaded from https://github.com/PINE-Lab/HAPPE.git. The downloaded HAPPE-master directory contains the required EEGLAB distribution, plugins, scripts, and supporting functions; separate installation of EEGLAB or MST1.0 is therefore not required.
iv. *Configuring MATLAB paths:* To add the necessary HAPPE directories to the MATLAB search path and configure the MATLAB environment, users should follow the instructions on pages 10–15 of the HAPPE User Guide included in the HAPPE-master directory. To avoid dependency conflicts, previously installed versions of EEGLAB or MST1.0 should not be simultaneously present on the MATLAB path.
v. *EEG pre-processing*: After completing the setup, users can preprocess the tutorial.raw EEG files using the preprocessing parameters provided in the accompanying Zenodo repository.^1^ ^2^ The resulting preprocessed EEG datasets are saved in.set format in the designated directory, together with the data-quality and HAPPE pipeline-quality metrics. Users may consult HAPPE manuscripts (Gabard-Durnam et al., 2018; Lopez et al., 2022; Monachino et al., 2022) and the user guide for example pipeline decisions and parameter recommendations if preprocessing their own EEG data instead of the tutorial files.
vi. *Executing microstate analysis*: Microstate analysis can be run in batch mode on EEG files stored in the designated directory by entering “generateMicroStates” in the MATLAB Command Window. Command prompts will follow to guide the user through analysis decision points.

Below, we provide a comprehensive guide to using the *generateMicroStates.m* script, describing parameter choices and quality checks relevant for pediatric EEG datasets.

### 7.1 Preprocessing requirements

The first preprocessing requirement is that all input EEG files must have consistent sampling rates (datasets used in this tutorial have a sampling rate of 1,000 Hz) and the number of channels across participants/files. Previous microstate studies on the adult population (Kleinert et al., 2023; Liu et al., 2020; Khanna et al., 2014) suggest a minimum requirement of having approximately 2 min of usable data from a participant (challenging for developmental populations, though see Bagdasarov et al., 2024a) to obtain stable microstate maps with ∼20 to 50 participants. In our experience with running the HAPPE analysis pipeline on infant datasets, including data subsets selected based on usable data lengths to examine this point specifically, we found that each EEG preprocessed file should contain at least 30 s of usable data to obtain reliable microstate maps with at least 20 participants. Analysis performed on recordings shorter than 30 s of developmental data from different age groups yielded unstable microstate maps. In accordance with previous developmental literature (Bagdasarov et al., 2024b) and the reliability of results in our datasets, we broadband filter our data between 1 and 40 Hz for resting state and 0.1–40 Hz for task condition before microstate analysis (band-specific analysis can be included if the research question requires it). Users are recommended to use average-referenced datasets for all microstate computations to reduce any reference bias (Poulsen et al., 2018). After ensuring that the preprocessed EEG data fulfills these required criteria, our automated generateMicroState.m script is ready to run. If using HAPPE for preprocessing, users can check reports with data and pipeline quality metrics to set additional data quality thresholds (e.g., files with more than a certain number of interpolated channels) to report in manuscripts for transparency. In the following sub-sections, we provide stepwise details and the functions involved in the execution pipeline of generateMicroState.m.

### 7.2 Script setup and environment initialization

Upon execution, the generateMicroState.m script:

- Initializes paths to internal HAPPE subdirectories and EEGLAB plugins (including MST1.0). We recommend downloading MST1.0 toolbox from our Github repository (link here) which comes with a slight modification to auto-save solution optimization figures while running the script.
- Prompts the user to manually input the path to the directory that contains the preprocessed.set files.
- Automatically creates an output folder *generateMicrostates* within that directory to store all results and figures.

### 7.3 Selecting the number of microstates

The users can choose how they want to determine the number of microstate classes. The script interactively walks the users via a command-line prompt to select the number of microstates (with an option to bypass the interactivity). They are provided with the following options:

- Preset (P): Users can manually specify the number of microstates (e.g., 4 for canonical A-D classes).
- Interactive (I): The script runs model selection across a specified range (2–8) and presents CV error and GEV plots to guide users’ decisions.

Recommendation: For developmental datasets where prior assumptions at different ages are not yet clear, interactive model selection (I) is preferable. The script is automated after this point to facilitate efficient, reproducible microstate analysis across large developmental EEG datasets. Although the following steps are automated within HAPPE, we provide annotations and justifications for the code below to allow users who wish to get “under the hood” and deeply understand the method.

### 7.4 GFP peak selection for clustering

Microstates are extracted at points of high Global Field Power (GFP), reflecting moments of topographic stability. These GFP maps are stored and later clustered to identify recurring spatial patterns. The code to extract GFP maps is provided below:

[EEG, ALLEEG] = pop_micro_selectdata (…“Npeaks,” 1,000, “GFPthresh,” 1,…)

Parameters used:

- “Npeaks”: Defines the number of GFP peaks extracted from the EEG recording. We typically use Npeaks = 1,000 (recommended) to ensure adequate sampling of GFP peaks. Although using Npeaks = 500 substantially reduced computation time and did not produce observable differences in the younger infant dataset, the resulting topographic maps were unreliable for the older infant datasets.
- “GFPthresh”: We typically use GFPthresh = 1, where maps with GFP exceeding 1 times the standard deviation of GFPs are rejected. Outliers beyond this threshold often include artifacts of non-neural origin that can distort the microstate topographies.
- “MinPeakDist”: We use MinPeakDist = 10 to ensure a minimum temporal separation of 10 ms between peaks. This distance was chosen based on electrophysiological plausibility; also, increasing the MinPeakDist up to 50 did not substantially improve processing speed.

### 7.5 Microstate segmentation via modified K-means

Clustering of the GFP maps is performed using a *modkmeans* algorithm, which is a modified version of k-means clustering that ignores the polarity of EEG topography and models the strength of microstate activation at a given time-point (Pascual-Marqui et al., 1995).

EEG = pop_micro_segment (EEG, “algorithm,” “modkmeans,” “sorting,” “Global explained variance,” “Nmicrostates,” 2:8, “Nrepetitions,” 50, “max_iterations,” 1,000, “fitmeas,” “CV”)

This produces the prototypes of microstate template maps. The parameters used are:

- sorting = “Global explained variance” or GEV. This sorts the resulting maps by decreasing explained variance within the range of Nmicrostates provided.
- Nmicrostates = 2:8; this parameter recommendation specifies the range of clusters considered for model selection, chosen based on the total number of clusters repeatedly reported in the developmental and broader EEG microstate literature.
- Nrepetitions = 50; this parameter recommendation defines the number of times the stochastic clustering algorithm is restarted to evaluate multiple segmentations of the same dataset and select the best restart based on e.g., GEV. This recommendation is based on our test-retest analyses that indicated inconsistent solutions with fewer than 50 restarts. Increasing this value may further improve the robustness of the obtained solutions, although at the expense of an increased computational cost.
- max_iterations = 1,000; this parameter recommendation means that the algorithm keeps updating the clustering solution until convergence is reached or if it is not reached by 1,000 iterations, the algorithm forcibly stops after completing 1,000 iterations. We used 1,000 iterations to balance computational efficiency and solution stability.
- fitmeas = “CV.” In cross-validation (CV), the EEG data are split into a training set (e.g., 90% of the data) to estimate the microstate clusters and a separate test set to evaluate how well these clusters generalize to unseen data.

### 7.6 Template evaluation and selection

If the interactive mode was selected at the beginning, the script visualizes the cluster prototypes and computes the GEV vs. CV criterion goodness-of-fit plot to determine the optimal number of microstate classes. The cross-validation (CV) criterion implemented here follows Pascual-Marqui et al. (1995) and should not be confused with conventional held-out cross-validation used in the previous section with “fitmeas” that involved partitioning participants or EEG samples into training and testing sets. The CV criterion on the other hand provides an internal index for comparing clustering solutions and selecting the number of microstate classes, rather than a direct estimate of generalization to unseen participants or EEG data.

EEG = pop_micro_selectNmicro_local (EEG, “Measures,” {“CV,” “GEV”},…)

GEV quantifies how well the EEG topographies match their assigned microstate templates, weighted by Global Field Power (GFP), as shown in Equation 1:


$${\text{GEV}}_{\text{n}}=\text{Corr}\,{\left({\text{x}}_{\text{n}},{\text{a}}_{\text{n}}\right)}^{2}.\frac{{\text{GFP}}_{\text{n}}^{2}}{\sum_{{\text{n}}^{\prime }}{\text{GFP}}_{\text{n}\prime }^{2}}$$ (1)

where *x_n_* is the microstate map at time n, *a_n_* is the assigned microstate, and *GEV*_*n*_ is the global field power at time n. GEV tends to increase with more clusters, so the decision involves balancing improved fit against interpretability for which we use the CV criterion that penalizes model complexity, as shown in Equation 2:


$$\text{CV}=\sigma^{2}.{\left(\frac{C-1}{\text{C–K}-1}\right)}^{2}$$ (2)

where *C* is the number of maps (GFP peaks), *K* is the number of clusters (microstate classes), and *σ^2^* is the residual noise estimate. Both GEV and CV metrics are jointly considered in determining the optimal number of cluster solutions.

*Note: If the number of microstate classes were pre-selected at the beginning itself, the user can no longer visualize the measures-of-fit plot and will obtain the pre-determined number of microstates at the end of script execution (e.g., 4).*

### 7.7 Backfitting and smoothing

In the back-fitting procedure, each subject’s EEG dataset is scanned, and each time point is assigned a microstate class whose prototype map shows the highest spatial similarity (ignoring polarity) to the instantaneous map, thereby assigning microstate labels to each subject’s EEG recording using global map dissimilarity (Murray et al., 2008).

EEG = pop_micro_fit (EEG, “polarity,” 0)

To reduce spurious labeling, the algorithm repeatedly scans through the microstate segments and changes the label of time frames corresponding to short periods of unstable EEG topographies to the next most likely microstate class using the process of temporal smoothing (Poulsen et al., 2018). This is done until no microstate segment is smaller than the recommended threshold of 30 ms, which is set based on the general practice in the field but users may also use 20 ms as in Zhang et al. (2025).

EEG = pop_micro_smooth (EEG, “minTime,” 30, “smooth_ type,” “reject segments”)

### 7.8 Microstate metric extraction

The script computes multiple metrics per subject, including the *Global explained variance or GEV* (variance accounted for by each microstate class), *Occurrence* (average number of times a microstate is dominant during the EEG recording, measured as events per second), *Duration* (average duration for which a microstate is dominant during the EEG recording, measured in milliseconds), *Coverage* (percentage of total EEG recording during which a given microstate class is dominant), *Transition Probability or TP* (quantifying how frequently microstate of a certain class is followed by microstate of another class without correcting for the number of occurrences) matrices:

EEG = pop_micro_stats (EEG, “label_type,” “backfit”)

Each subject’s metrics are produced as a structured.mat file and saved as a.csv summary file in the *generateMicrostates* folder within the current directory.

### 7.9 Visualization and illustration

After full execution, the script automatically plots and saves:

- The microstate prototype topographies (2–8) of each microstate segmentation, sorted by decreasing GEV as *Nmicrostates.fig*.
- The measures-of-fit plot as *GEVvsCV.fig* (if interactive mode was selected).
- Plots and saves the GFP with microstate labels overlaid for the first subject as *microstatesGFP.fig*.

These plots provide the visualization of segmentation quality and microstate map interpretability.

## 8 Implementation using developmental datasets

We provide illustrative examples of microstate computation from task and resting-state EEG recordings across two pediatric age groups, with the raw EEG data (.raw format) and preprocessing parameters freely available at Zenodo (see text footnote 1). Specifically, we provide a subset of EEG files used in our previous studies (Ghosh et al., 2025; Forest et al., 2025). The following.*raw* EEG files can be found in the repository: 25 baseline EEG files each from 6-month-olds (***1***_191_********_6_********_*.raw) and 24-month-olds (***1***_191_********_24_********_*.raw), and 25 auditory task EEG files from 24-month-olds (***4***_191_********_24_********_*.raw). All codes used to process the datasets can be accessed from our GitHub repository,^4^ and all other preprocessing parameters used to process the raw EEG files using HAPPE_v4 have been made available on Zenodo (see text footnote 1). Separate input parameters were used to process the raw EEG corresponding to task (*inputParameters_task.mat*) and resting state (*inputParameters_rest.mat*) conditions, while the same generateMicroStates.m script was run on the generated HAPPE preprocessed files (*.set*) for computing microstates across all conditions. A comprehensive schematic outlining all steps used to obtain microstates from raw EEG data files has been presented as Figure 1.

**FIGURE 1:**
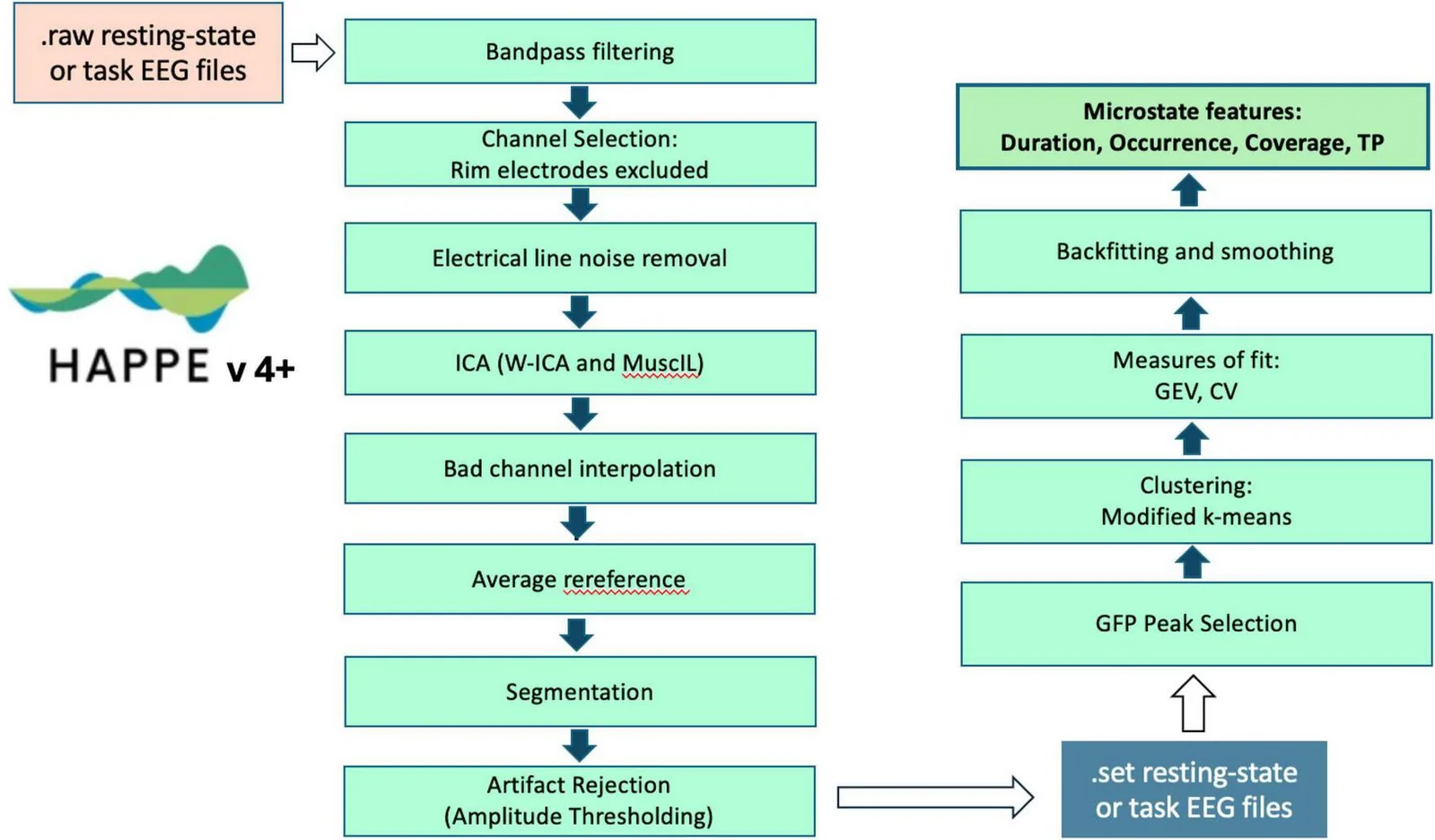
Generate microstates. Flow chart depicting the steps used to obtain microstate classes and their parameters from raw EEG data.

### 8.1 Participants

Our example data comes from a subsample of infants from Cape Town, South Africa recruited as part of the longitudinal Khula study which was designed to examine brain development as it relates to the development of executive functions in children during their first 1,000 days of life. Descriptions of recruitment procedures and other demographic details are published elsewhere (Ghosh et al., 2025; Forest et al., 2025; Zieff et al., 2024). All procedures were approved by the Human Research Ethics Committee at the University of Cape Town in South Africa and written consents were obtained from the caregivers.

### 8.2 EEG data acquisition

EEG data were acquired using the same protocol for the resting-state and task conditions. Infants sat on their caregiver’s lap in a quiet and dimly lit room during EEG recordings using high-density (128-channel) HydroCel Geodesic Sensor Nets (Magstim EGI, Whitland, United Kingdom). Data were collected at a sampling rate of 1,000 Hz, online referenced to the vertex (electrode Cz) via NetStation 5.4 software and impedances were maintained below 100 KΩ. For the two baseline conditions, the infants passively viewed a 3-min long silent screensaver-like video of moving shapes as used in Fox et al. (2024). For the auditory task condition, a 7-min long statistical learning task was presented. To keep the kids engaged, a silent video was played on the screen while the audio stimuli played. The task consisted of a series of 12 different pure tones, each 550 ms long with no inter-tone interval. The tones were grouped into four sets of triplets (like F^#^A^#^D, CG^#^C^#^, EGD^#^, etc.) and within a triplet, the tones were always presented in a predictable order (i.e., in F^#^A^#^D, F^#^ always preceded A^#^, and A^#^ always preceded D) but the presentation order of the triplets were randomized (F^#^A^#^D could be followed by CG^#^C^#^ or EGD^#^). Additionally, three triplets were presented twice more frequently than the fourth triplet as part of the global statistical structure. For further details on the statistical learning task, please refer to Forest et al. (2025).

### 8.3 Optimized preprocessing parameters across task and resting state

EEG.raw files from resting-state and task conditions were preprocessed using the Harvard Automated Processing Pipeline for EEG (HAPPE), open-source software optimized for processing infant EEG data (HAPPE and HAPPE+ER; Gabard-Durnam et al., 2018; Monachino et al., 2022). We preprocessed the.raw EEG files using HAPPE software version 4 on MATLAB (2022b) with EEGLAB toolbox (Delorme and Makeig, 2004). Preprocessing parameter files are provided on Zenodo. All the outer rim and eye electrodes of the net (electrodes E125, E126, E127, E128, E48, E119, E43, E49, E56, E63, E68, E73, E81, E88, E94, E99, E107, E113, E120, E44, E38, E32, E25, E21, E14, E8, E1, E121, E114, and E17) were removed from preprocessing because in developmental populations these electrodes are highly contaminated with artifacts (see Monachino et al., 2022). Electrical line noise at 50 Hz was removed using a notch filter between 45 and 55 Hz (Mitra and Pesaran, 1999), while data were bandpass filtered (using FIR) between 1 and 40 Hz for resting state and 0.1 and 40 Hz for task condition. To minimize artifacts within segments, data were corrected via wavelet-thresholding and channels whose probability was more than 3 standard deviations from the mean were detected as bad channels and were further interpolated via spherical spline interpolation. Different participants could have different bad channels in their data that got interpolated individually. Other EEG preprocessing pipelines that use only independent component analysis (ICA) for artifact removal undergo high rates of IC rejection in developmental EEG datasets and rely on methods that consider whole-scalp information at once, increasing the potential of altering and rejecting meaningful largescale spatial information required for microstate computations. The additional step of wavelet thresholding within HAPPE during preprocessing is less likely to over-remove signal and alter global spatial topographies because it operates channel-independently with precision in temporal and frequency domains. Therefore, using HAPPE for preprocessing EEG datasets prior to microstate analysis has a clear advantage.

The data were finally average re-referenced after they were segmented into short epochs. For the resting-state condition, the continuous EEG recording from each participant was epoched into 2,000-ms-long segments and any segments with remaining artifact were removed using an amplitude-based cutoff of ± 150 μV. For the auditory task condition, epochs were segmented based on the stimulus onset tags (“st11” and “st21”). 900 ms long segments post stimulus onset (0 ms) were epoched and any further artifact-laden segments were removed using amplitude-based cutoff of ± 200 μV. No baseline correction was performed. For each raw task input file, preprocessing generated three.set output files. The first.set file contained segments only with onset condition “st11,” second.set file contained segments only with onset condition “st21” and the third.set file contained segments from both the onset conditions. Because this tutorial did not aim to compare condition-specific microstate differences, we used the.set file containing both onset conditions. Although the EEG data from both conditions were segmented into shorter epochs during preprocessing, all eligible trials from each participant were concatenated in time before microstate analysis, enabling a larger and more representative pool of GFP-peak maps for stable microstate estimation.

### 8.4 Infant microstates: outputs

EEG microstate analysis offers a spatio-temporal characterization of large-scale brain network activity where the spatial estimates are indexed by the topographies of the microstate classes (i.e., the spatial configuration of the changing scalp electric field) and the statistical features of the individual microstate classes. Both these properties may change with age and task conditions, as would the underlying large-scale brain network activity, much more rapidly in the developmental population. Our automated script produces the following outputs while computing microstates: Nmicrostates.fig, GEVvsCV.fig, microstatesGFP.fig and generateMicrostate_outputs.csv. All the output files can be found in the generateMicrostates folder. To facilitate understanding of these outputs amongst users, we provide a stepwise walkthrough of the analysis outputs using the publicly available developmental dataset from the following three conditions.

#### 8.4.1 Resting state condition at 6 months

Using a polarity-invariant modified k-means clustering method and other parameters as mentioned in section 4, the microstates are first quantified into prototypes for a specified range of clusters. We use a reasonable range of 2–8 prototype topographies, which have been presented in Figure 2a and are saved as *Nmicrostates.fig* in the *generateMicrostates* folder. By default, the prototype topographies are sorted by their decreasing Global Explained Variances.

**FIGURE 2:**
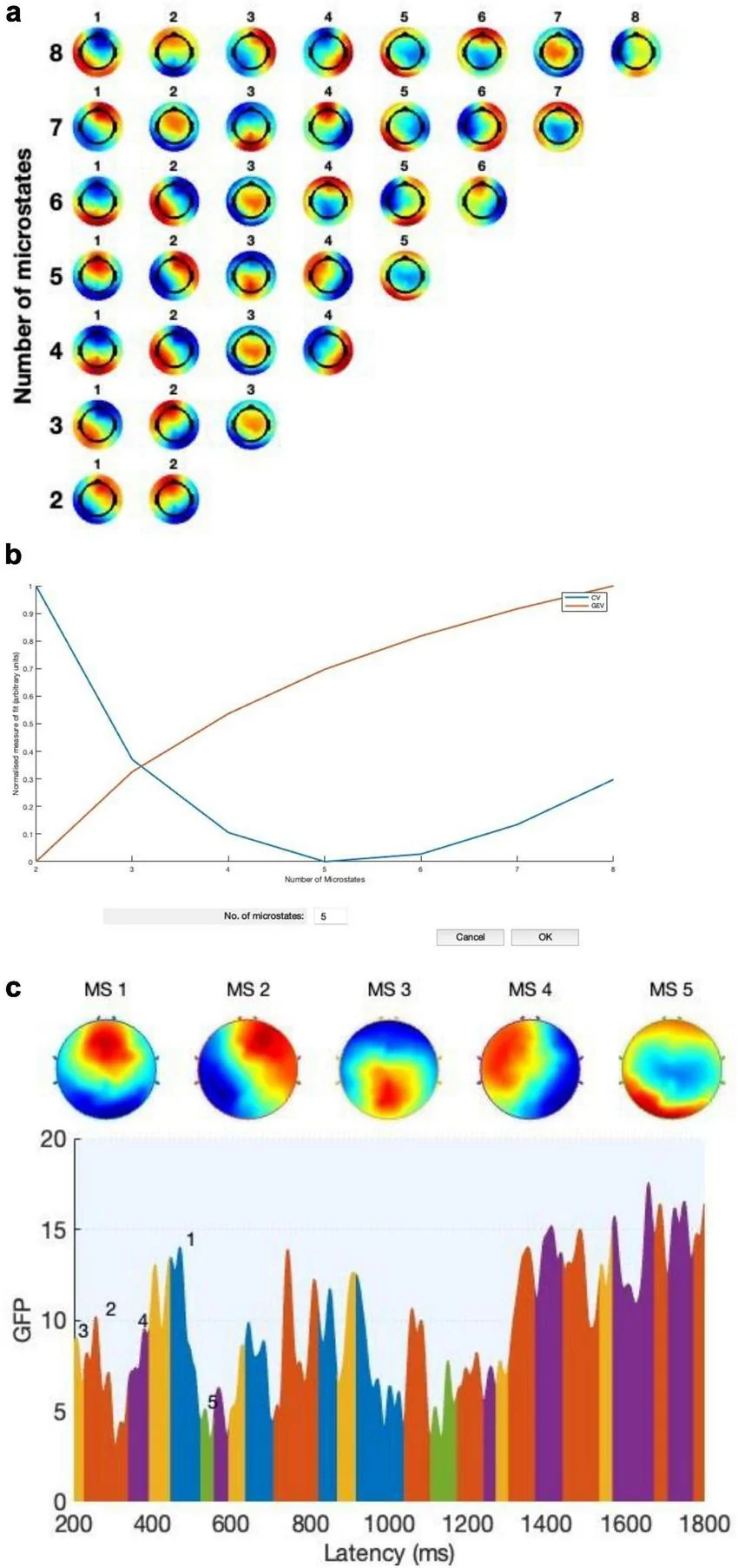
**(a)** Microstate prototypes for the 6M resting-state condition. The figure represents 2–8 microstate prototype topographies (i.e., cluster configurations) sorted by their GEVs for resting-state condition at 6 months. **(b)** Plot for determining the optimum number of microstate solutions for 6M resting-state condition. Normalized GEV vs. CV plot showing the optimal number of microstate solutions for resting state at 6 months condition. **(c)** Dominant microstates across time at the 6M resting state condition. The figure represents the Global Field Power (GFP) of the concatenated time series from a subject, annotated for the active microstate classes (MS1, MS2, MS3, MS4, and MS5) in the time window from 200 to 1,800 ms for the resting state-condition at 6 months.

The next step involves a qualitative assessment of these microstate prototypes for class number determination which is often a trade-off between the goodness-of-fit and complexity introduced by a larger number of clusters. If the users select an interactive mode to determine the number of microstate classes at the beginning of the analysis (recommended), a plot of the normalized GEV and CV measures gets automatically saved as *GEVvsCV.fig* in the *generateMicrostates* folder. The algorithm converged at a clustering solution of 5 classes for the resting state at 6-month condition dataset, as seen in Figure 2b.

Once the optimum clustering solution is obtained, the clusters are fitted back to the individual EEG datasets of this group condition. A figure corresponding to the backfitted data on subject 1, called *microstatesGFP.fig* gets saved in the *generateMicrostates* folder. Note: While microstate metrics in infancy are still emerging, prior work has demonstrated that four to seven microstates typically account for the majority of topographic variance even in infants and toddlers.

#### 8.4.2 Resting state condition at 24 months

The same segmentation parameters as the 6-month resting state were used for this condition, with a range of 2–8 cluster prototypes generated as *Nmicrostates.fig* and presented here as Figure 3a.

**FIGURE 3:**
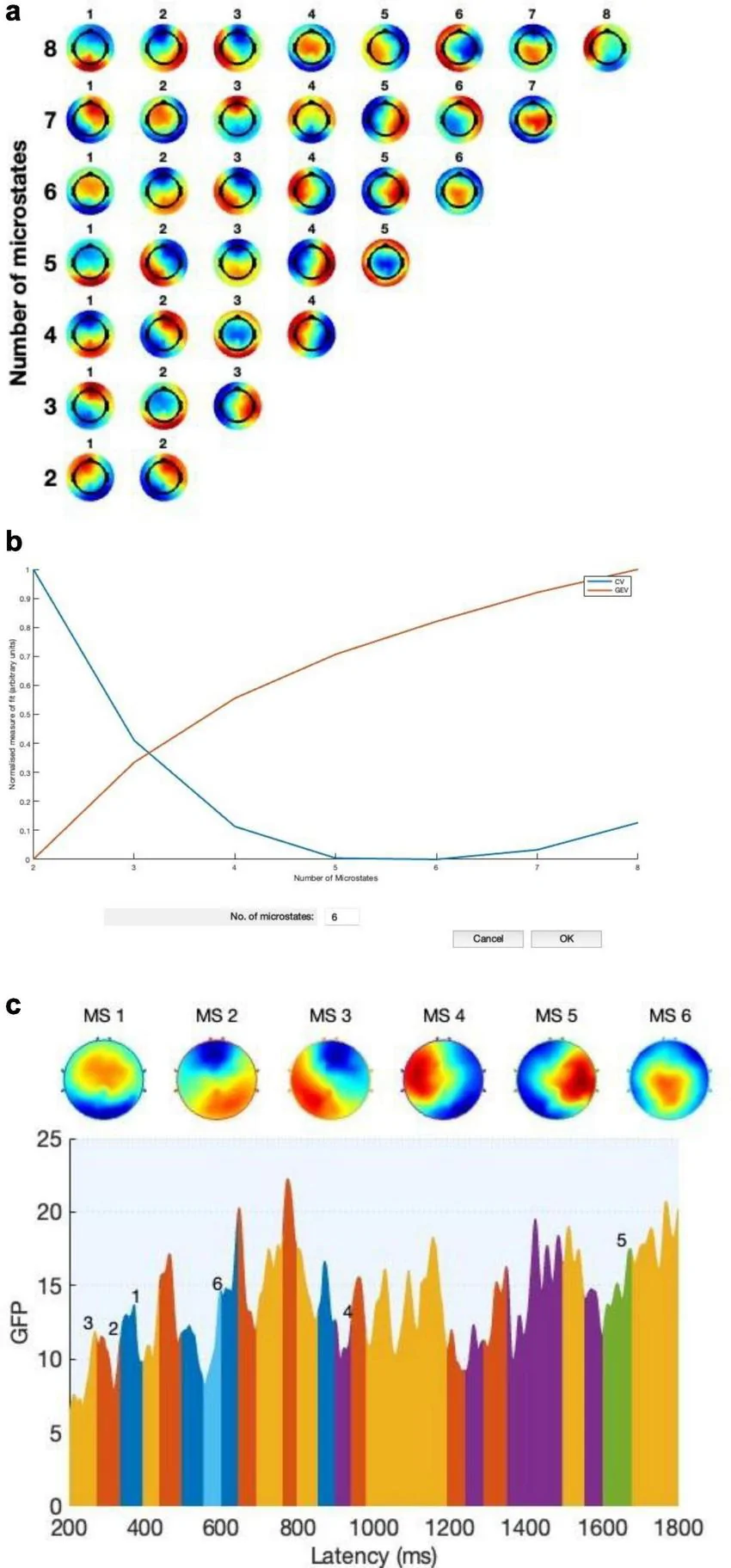
**(a)** Microstate prototypes for the 24M resting-state condition. The figure represents 2–8 microstate prototype topographies (i.e., cluster configurations) sorted by their GEVs for the resting-state condition at 24 months. **(b)** Plot for determining the optimum number of microstate solutions for 24M resting-state condition. Normalized GEV vs. CV plot showing the optimal number of microstate solutions for resting state at 24 months condition. **(c)** Dominant microstates across time at 24M resting state condition. The figure represents the Global Field Power (GFP) of the concatenated time series from a subject, annotated for the active microstate classes (MS1, MS2, MS3, MS4, MS5, and MS6) in the time window from 200 to 1,800 ms for the resting state-condition at 24 months.

Next, we let the algorithm pick the optimum number of classes based on GEV and CV measures, but the CV plot in this case was a flat line between 5 and 6 solutions (Figure 3b). To validate whether multiple stochastic runs of the algorithm (involving multiple restarts) could improve the model performance, we computed the microstate solutions three more times but the model’s optimal fit stayed between 5 and 6 classes. Still, we recommend repeated solution computations (between 3 and 5 runs to balance computational burden against potential for solution change) as the first approach to resolving ambiguous class thresholds based on GEV and CV criterion. In this case, given the additional solution computations did not clarify the class threshold, we used a combination of additional evidence to decide on 6 classes: (a) we referenced prior developmental literature (Ghosh et al., 2025) that identified 6 solutions for this age, (b) we observed that relative to the five-class solution, the six-class solution produced clearer topological matches to existing microstates (compared against developmental and adult microstate solutions), and (c) we observed a higher GEV value for the six-class solution indicating more variance explained in the data (this is generally expected as class number increases but should be checked all the same). Note: The data from 25 participants used here is a subsample of data used in a previous study (Ghosh et al., 2025) with > 200 participants, for which the algorithm clearly converged at 6 solutions.

Finally, the GFP plot with the active microstate classes for subject 1’s resting state EEG dataset at 24 months was generated with the 6 microstate solutions (Figure 3c) as a representative example.

#### 8.4.3 Auditory task condition at 24 months

To ensure comparability of microstate features across conditions, we used identical clustering parameters for both resting-state and auditory task conditions. Using the same parameters allows the resulting microstate maps to be interpreted within a common representational space, useful for standardization and minimization of confounds introduced by methodological variability. We used a range of 2–8 clusters with the modified k-means algorithm (Figure 4a), ensuring observed differences between conditions reflect underlying neurophysiological changes rather than parameter-driven artifacts (Michel and Koenig, 2018; Khanna et al., 2015). Moreover, several studies have demonstrated that the canonical microstate classes (A–D) and their associated neural generators remain broadly consistent across resting and task conditions (Milz et al., 2016; Seitzman et al., 2017).

**FIGURE 4:**
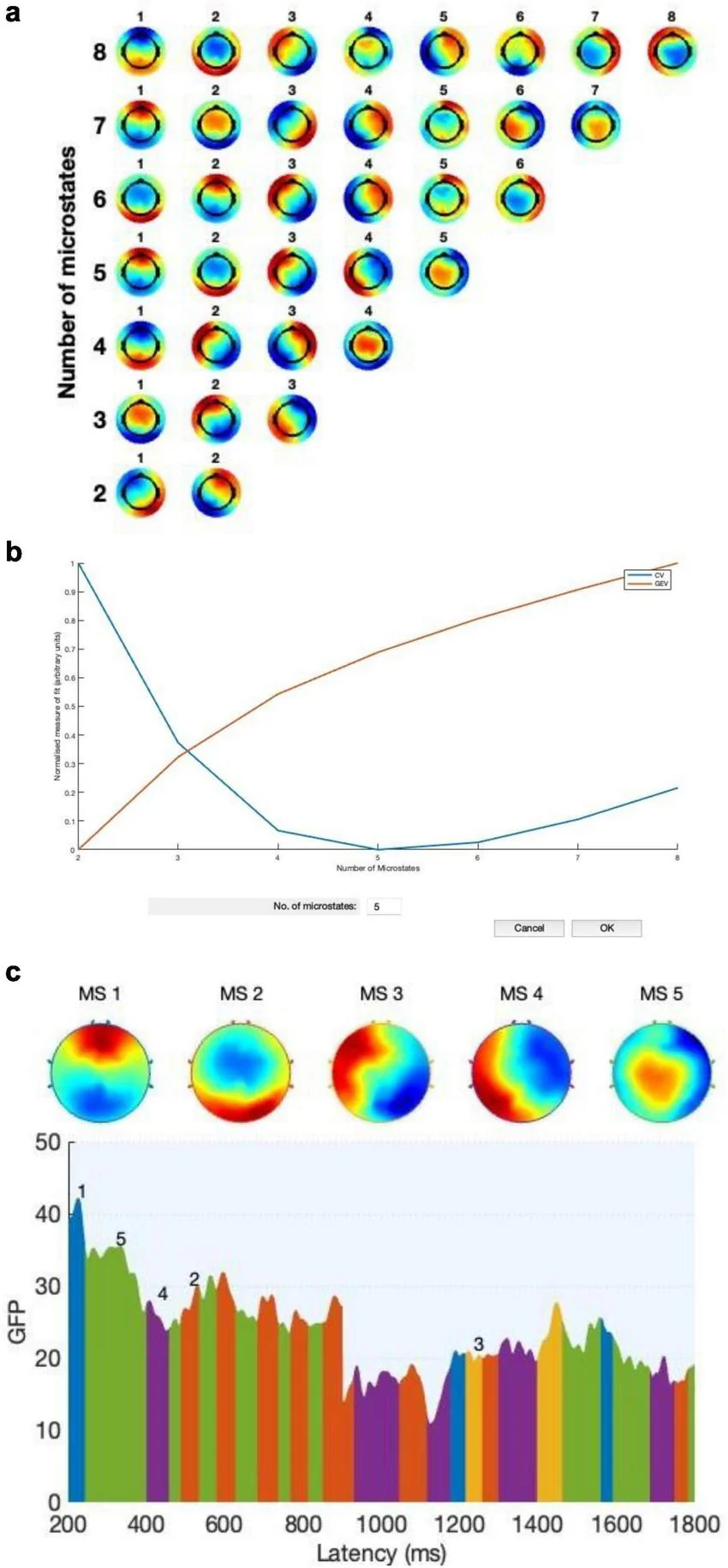
**(a)** Microstate prototypes for 24M auditory-task condition. The figure represents 2–8 microstate prototype topographies (i.e., cluster configurations) sorted by their GEVs for the auditory statistical learning task condition at 24 months. **(b)** Plot for determining the optimum number of microstate solutions for 24M task condition. Normalized GEV vs. CV plot showing the optimal number of microstate solutions for auditory task at 24 months condition. **(c)** Dominant microstates across time at 24M auditory task condition. The figure represents the Global Field Power (GFP) of the concatenated time series from a subject, annotated for the active microstate classes (MS1, MS2, MS3, MS4 and MS5) in the time window from 200 to 1,800 ms for the auditory statistical learning task condition at 24 months. Note: 200–1,800 ms post-stimulus window was selected for visualization purposes only.

In developmental EEG, where maturational effects already introduce significant variability in topography and signal-to-noise (Bagdasarov et al., 2024a; Koenig et al., 2002; Tomescu et al., 2024), using a unified clustering framework enables a fair comparison of microstate classes and their features. We used the CV criterion to penalize the complexity associated with a larger number of clusters in both resting state and task conditions. This prevented overfitting while the best clustering solution was selected in a data-driven manner based on the GEV values. For this group, the solution clearly converged to 5 classes as seen in Figure 4b.

Next, the 5 microstate classes are back-fitted to the individual EEG datasets of the 25 participants from the auditory task-condition. Figure 4c shows the GFP series annotated with the microstate classes for subject 1 between 200 and 1,800 ms after task onset (0 ms).

### 8.5 Extracting microstate classes and their statistical features

The topographies obtained from the plots in *microstatesGFP.fig* in all three conditions were relabeled from a numerical (MS1, MS2, etc.) to alphabetical (MS.A, MS.B, etc.) naming scheme as is more commonly used in the microstate literature (Koenig et al., 2002; Michel and Koenig, 2018). To maintain consistency between the spatial topographies and microstate labels across conditions, the labels were manually adjusted across conditions through visual inspection. We used the reference of topographies (polarity-invariant) in Tarailis et al., 2024 (currently one of the most comprehensive and widely cited frameworks for EEG microstate classification) such that spatial topography with a right-frontal left-posterior would be classified as microstate class “A,” that with a left-frontal right-posterior would be classified as class “B,” horizontally symmetric anterior-to-posterior as class “C,” fronto-central as class “D,” centro-parietal maximum as class “E,” left-lateralized maximum as class “F,” and right-lateralized maximum as class “G.” Finally, the microstate labels were accordingly adjusted in the.csv files (*generateMicrostate_outputs.csv)* for the corresponding microstate classes. In our example dataset, the labels were renamed in the following manner across conditions:

(A) Resting-state (6 months): MS.1- > MS.C; MS.2- > MS. A; MS.3- > MS.E; MS.4- > MS.B; MS.5- > MS.D

(B) Resting-state (24 months): MS.1- > MS.D; MS.2- > MS.B; MS.3- > MS.A; MS.4- > MS.F; MS.5- > MS.G; MS.6- > MS.E

(C) Auditory task (24 months): MS.1- > MS.C; MS.2- > MS.D; MS.3- > MS.B; MS.4- > MS.A; MS.5- > MS.E

For publication and cross-study comparisons, we recommend using relabeled microstates (as shown in Figure 5).

**FIGURE 5:**
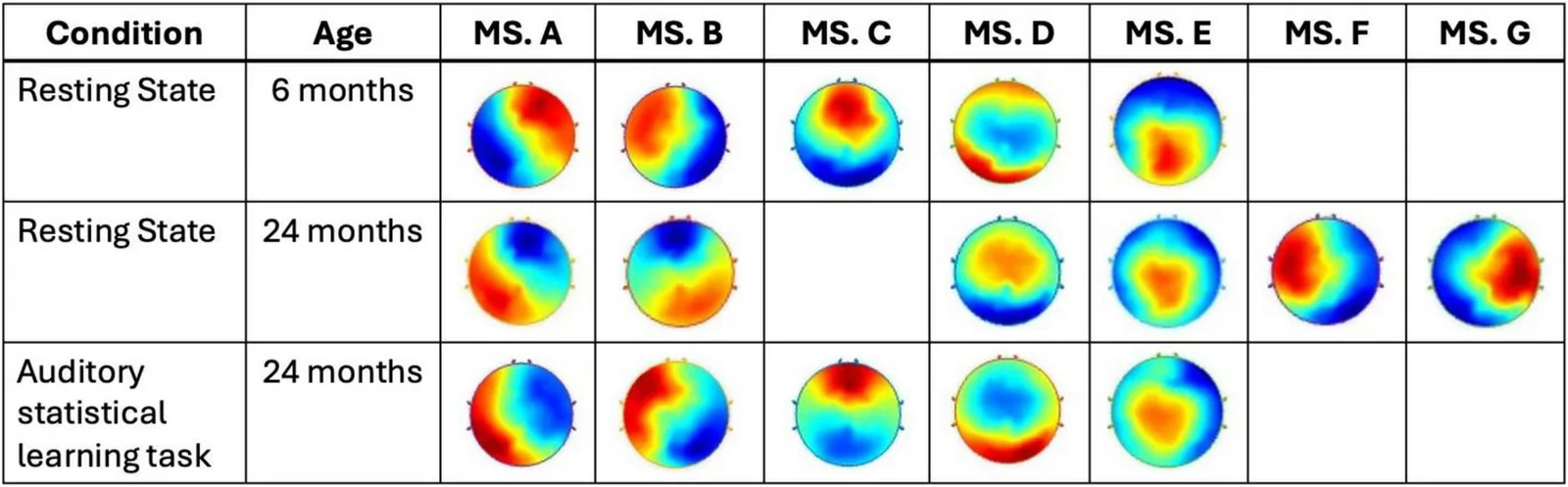
Microstates in resting state and task. The top-panel shows microstate topographies obtained for the 6-month-resting state condition; the second shows the microstate topographies obtained for the 24-month-resting state condition; the bottom-panel shows microstate topographies obtained for the 24-month-auditory statistical learning task condition.

Note that the Microstatelab toolbox (Nagabhushan Kalburgi et al., 2024) supports labeling of the grand mean microstate maps according to the overall highest spatial correlation with published adult template maps that contain the four to seven prototypical microstate classes identified by Koenig et al. (2002) and the seven microstate maps identified by Custo et al. (2017).

### 8.6 Visualization of microstate statistics

In this section, we present the descriptive statistics (in Tables 1, 2 and Figures 6–8) of the following microstate properties for all the conditions: (1) Durations, (2) Occurrences, and (3) Transition Probabilities. The generated.csv files with computed microstate statistics for each participant have been provided at Zenodo with filenames *stats_rest_6M.csv, stats_rest_24M_6sol.*csv and *stats_task_24M.csv.*

**TABLE 1:** Microstate durations.

Class	Rest 6M	Rest 24M	Task 24M
MS. A	79.51 (70.45–98.56)	74.18 (60.28–87.61)	78.32 (64.37–115.15)
MS. B	76.31 (69.58–94.45)	76.37 (66.75–88.81)	81.20 (67.00–138.30)
MS. C	92.20 (76.47–120.31)	–	91.92 (76.92–157.84)
MS. D	79.41 (61.17–103.08)	77.99 (65.48–101.06)	89.96 (71.09–124.58)
MS. E	80.16 (66.75–99.05)	66.34 (57.92–89.06)	74.35 (58.91–143.30)
MS. F	–	66.92 (58.88–83.85)	–
MS. G	–	64.98 (59.49–74.71)	–

The mean microstate durations across all 25 participants, along with the minimum and maximum duration values depicting the range for each microstate class, have been presented for the resting state condition at 6 months, resting state condition at 24 months, and the auditory task condition at 24 months.

**TABLE 2:** Microstate occurrences.

Class	Rest 6M	Rest 24M	Task 24M
MS. A	2.51 (1.78–3.22)	2.60 (1.48–3.40)	2.34 (1.54–3.03)
MS. B	2.33 (1.24–3.29)	2.71 (1.41–3.19)	2.40 (1.80–3.04)
MS. C	2.87 (2.29–3.58)	–	2.67 (0.95–3.51)
MS. D	2.18 (1.10–2.95)	2.74 (1.53–3.32)	2.66 (1.63–3.46)
MS. E	2.26 (1.55–2.85)	1.86 (0.77–2.80)	1.88 (1.07–2.46)
MS. F	–	2.04 (1.59–2.61)	–
MS. G	–	1.90 (1.29–2.32)	–

The mean microstate occurrences across all 25 participants, along with the minimum and maximum duration values depicting the range for each microstate class have been presented for the resting state condition at 6 months, resting state condition at 24 months, and the auditory task condition at 24 months.

**FIGURE 6:**
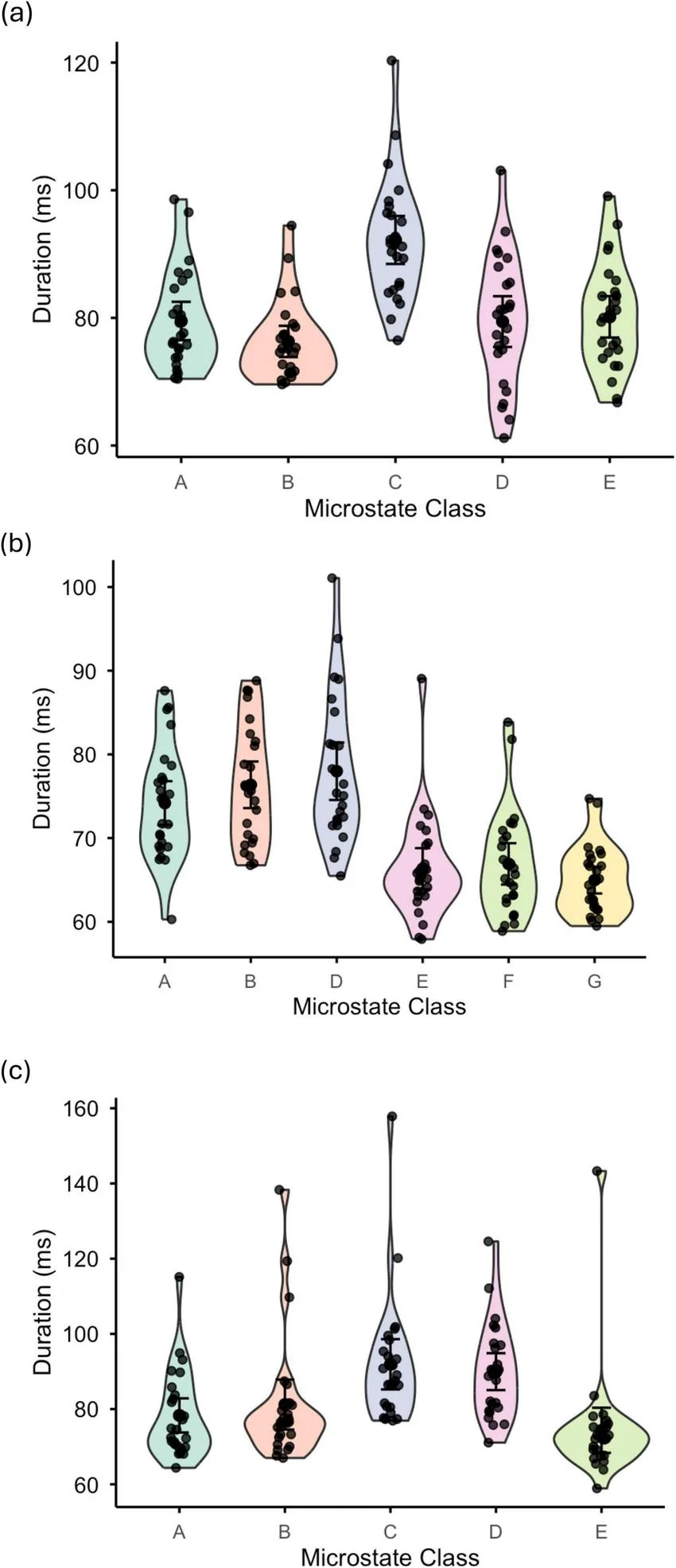
Microstate durations by age and condition: The violin plots represent class-wise distribution of microstate durations for the **(a)** resting state condition at 6 months, **(b)** resting state condition at 24 months, and **(c)** auditory task condition at 24 months.

**FIGURE 7:**
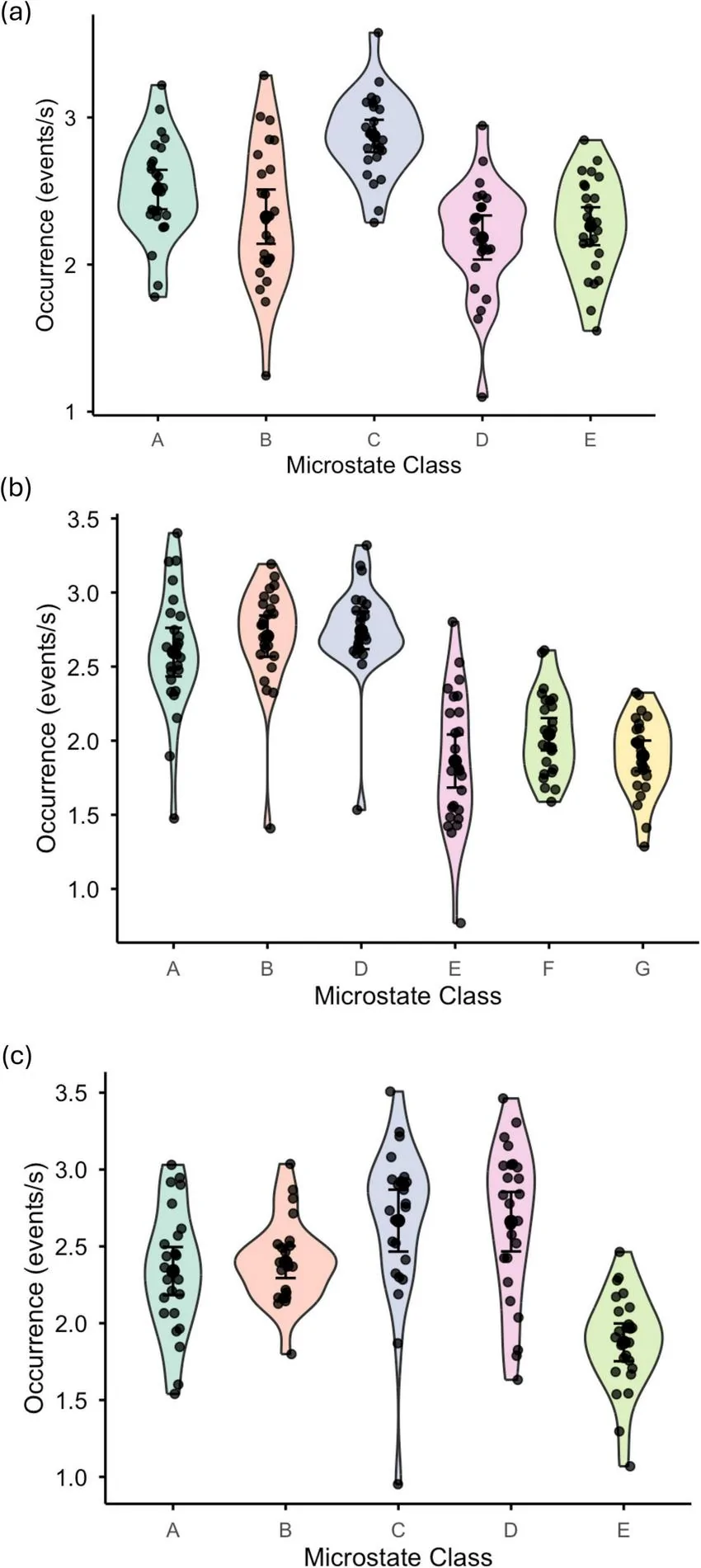
Microstate occurrences by age and condition: The violin plots represent class-wise distribution of microstate occurrences for the **(a)** resting state condition at 6 months, **(b)** resting state condition at 24 months, and **(c)** auditory task condition at 24 months.

**FIGURE 8:**
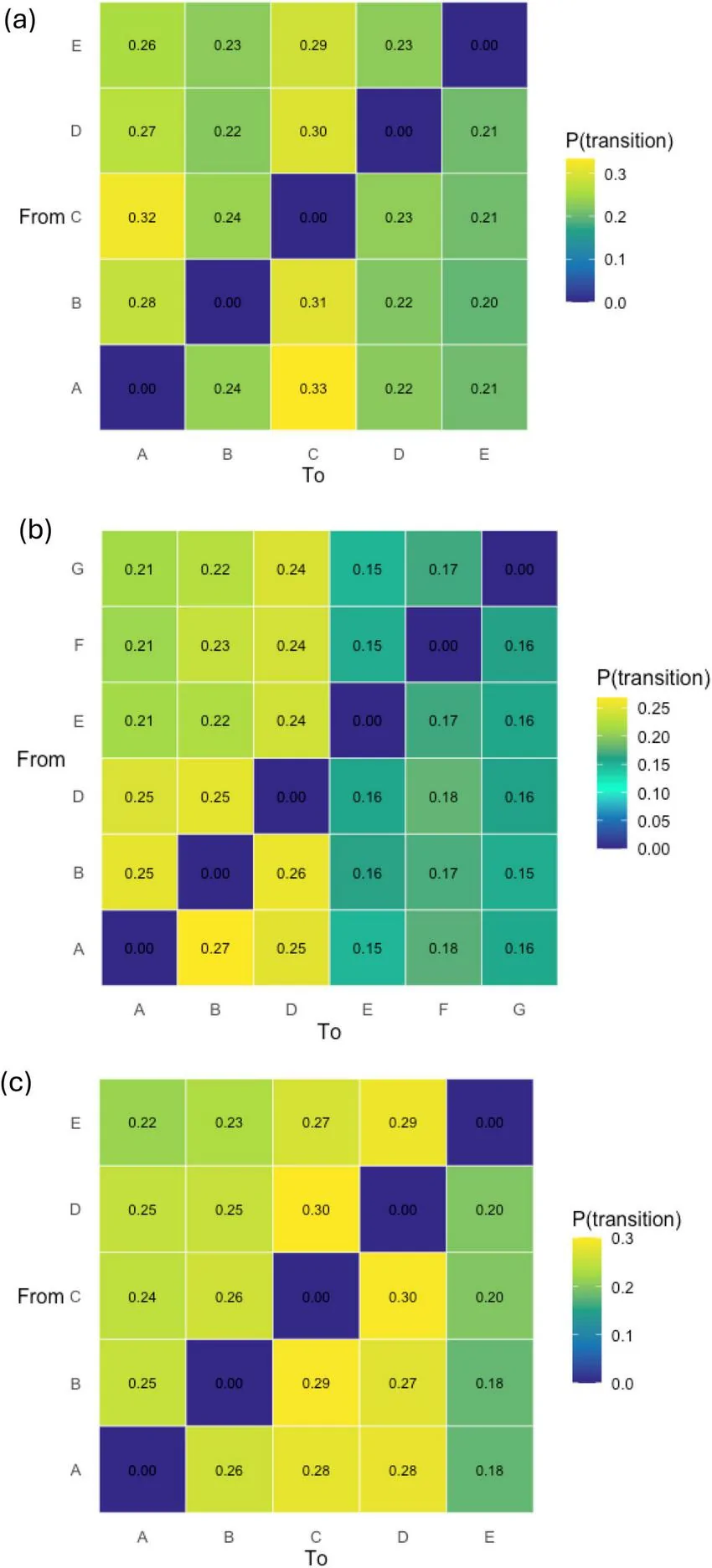
Microstate transition probabilities. The transition probability matrices illustrate the mean probability of transitioning from one microstate class (*y*-axis) to another microstate class (*x*-axis) for the **(a)** resting state condition at 6 months, **(b)** resting state condition at 24 months, and **(c)** auditory task condition at 24 months. Each tile represents the mean transition probability between a pair of microstates, averaged across all participants for each condition.

## 9 Discussion

An important aspect of our tutorial is the application of microstate analysis to HAPPE-preprocessed EEG data. HAPPE uses developmentally validated parameters to remove artifacts while retaining as much usable data as possible, which is particularly important for developmental datasets. Because microstate clustering depends on the spatial configuration of scalp potentials, residual artifacts and noisy channels can distort the resulting microstate topographies resulting in unreliable template maps. Preprocessing with HAPPE therefore provides an appropriate foundation for obtaining physiologically interpretable microstate maps. The HAPPE-microstate pipeline further reduces the number of user-dependent analytic decisions through a semi-automated, primarily data-driven workflow, while retaining the flexibility to specify a fixed number of microstates *a priori*. We have demonstrated the efficacy of our microstate computation on publicly made available datasets (part of which was is also replicated in a larger cohort in Ghosh et al., 2025) using developmentally optimized parameters as demonstrated in the previous section. An important objective of this tutorial is to provide an approach to the challenge of selecting an appropriate number of microstate maps in developmental populations, where normative templates remain limited. Many adult studies have fixed the number of classes a priori at four to facilitate comparison with the canonical microstates, sometimes without fully reporting the trade-off between model fit and complexity. Even in adult populations, when data-driven approaches to class count are performed, additional microstate classes are often identified beyond the canonical 4 classes, suggesting there is more to be learned about microstate class structures measured at any age. Given these circumstances, our tutorial guides researchers in deriving data-driven microstate solutions using global explained variance (GEV) and the CV criterion so that the selected solution best balances high explained variance, low residual noise, and topographic stability. We recommend taking this data-driven approach to obtain microstate class structure for developmental studies unless the project has an explicit goal to compare their microstates against either canonical adult or previously observed developmental solutions. In that case, we note that selecting the same number of microstate classes as in prior literature does not guarantee the same microstates will be observed. We instead recommend evaluating the data driven solutions and choosing the class solution that (1) provides the desired microstate topography(ies) for comparison and then (2) offers the GEV/CV values that best balance data fit amongst the class number options that include the desired topography(ies).

Many widely used microstate analysis pipelines use a two-stage clustering procedure: spatial clustering is first performed on trials separately for each participant to identify individual-level prototypes, followed by a second step involving clustering of these prototypes across participants to obtain group-level maps (Brunet et al., 2011; Nagabhushan Kalburgi et al., 2024). In contrast, the HAPPE-microstate pipeline samples GFP-peak maps from each participant and performs a single group-level spatial clustering of the pooled maps using polarity-invariant modified k-means (as described by Poulsen et al., 2018). This approach prevents the information loss introduced when group-level clustering operates on already-reduced individual-level prototypes. Individual-level clustering can be unreliable when recordings are short, noisy, or contain few usable GFP peaks, a common case in developmental EEG. Our approach also reduces the risk of participants with longer recordings contributing disproportionately to the prototype representations. The choice of data used to derive common templates requires careful consideration in studies involving two or more conditions. Seitzman et al. (2017), for example, derived group-level templates from pooled eyes-closed resting-state data and applied these templates to both resting-state and serial-subtraction task conditions. Although this approach ensures that identical templates are used across conditions, it may constrain task data to resting-state topographies and reduce sensitivity to task-specific configurations. Based on this and other findings in the adult literature (Gelormini et al., 2025; Chen et al., 2024; D’Croz-Baron et al., 2021), it remains unclear whether a common or condition-specific template renders more reliable microstate maps and subsequently microstate temporal dynamics. For direct group or condition comparisons, we recommend deriving a common template solution from a balanced pool in which participants and conditions contribute comparable amounts of data. Condition-specific clustering should additionally be conducted as a sensitivity analysis to identify potentially meaningful age-associated or task-specific topographies that might be obscured in the common maps. To facilitate decision-making while implementing microstate analyses across groups and conditions, we provide a flow-chart in Figure 9 to help researchers navigate the relevant methodological contingencies. Please note that we do not wish to be overly-prescriptive without knowing future study designs and aims as this is a very complicated issue (more broadly for developmental studies across a number of measures, not just microstates) without a singular solution.

**FIGURE 9:**
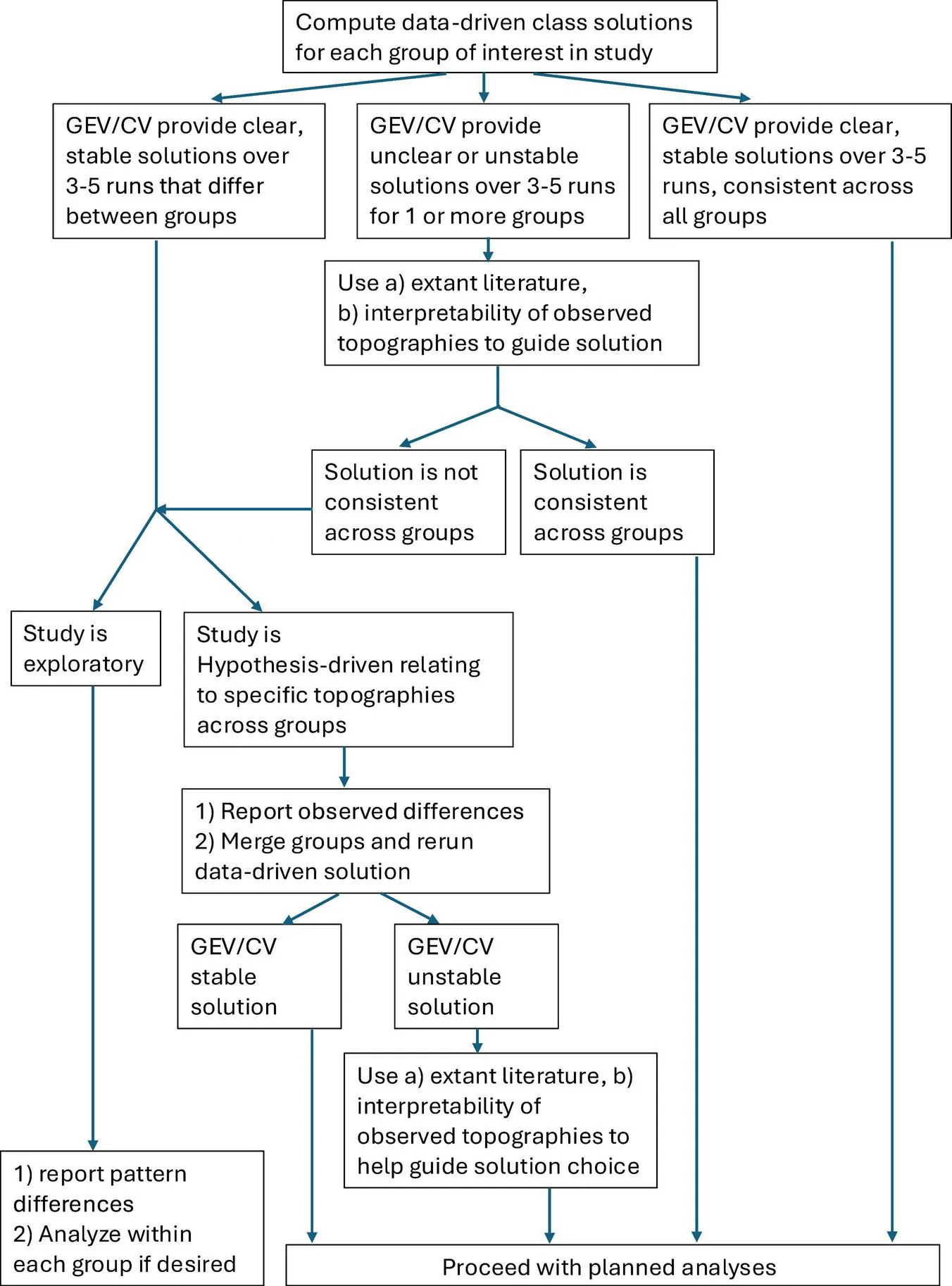
Decision tree guiding user decisions for EEG microstate analyses in developmental populations.

Although the HAPPE-microstate pipeline can analyze both resting-state and continuous task-based EEG, ERP (event-related potential) microstate analysis in event-related experimental designs is currently outside the scope of this tutorial. ERP microstate analysis represents a distinct approach in which instead of concatenating the trials of a participant in time (like in our demonstrated approach), the preprocessed trials are typically averaged within each participant and condition. The resulting participant-level grand-averaged ERPs are then submitted to topographic clustering, and the resulting templates are backfitted to participant-level ERPs (Koenig et al., 2013; Prete et al., 2022; Bagdasarov et al., 2024b). Condition-specific topographies can also be statistically compared using spatial correlation or topographic analysis of variance (Koenig et al., 2011). Unlike continuous task microstates which consist of the non-phase locked information from the EEG, ERP microstates characterize the timing and (potentially polarity-variant) topography of phase-locked evoked activity. In previous studies, Prete et al. (2022) performed clustering and back-fitting on participant- and condition-specific ERPs, but they did not mention how the ERPs were generated, at what time point they appeared or which peaks were considered as ERPs. Bagdasarov et al. (2024b) used visual inspection of the segmented grand-averaged data to identify the event-related microstate generated in response to a developmentally suited go/no-go paradigm before backfitting it to participant-level error-related activity. Though this might not be a suitable approach for large sample sizes, the authors note that future work should examine the relationship between ERPs measured during traditional windows of time compared to those derived from the results of microstate segmentation. No universally validated minimum epoch length for ERP microstates has been established for either developmental or adult ERP microstate analysis. Future researchers should report all factors contributing to clustering and evaluate template stability through repeated subsampling or split-half analyses. Additionally, the same prestimulus interval and baseline-correction procedure should be applied across conditions before ERP microstate analysis. Traditional baseline subtraction removes the mean voltage during the selected prestimulus interval from every post-stimulus time point at each electrode. Consequently, baseline activity and noise are propagated into the post-stimulus signal and can influence subsequent topographic comparisons (Alday, 2019). When microstate parameters are compared between sustained task and resting-state EEG (as demonstrated in this tutorial), baseline correction should generally not be applied only to the task condition while the resting-state data remain uncorrected, because this would introduce a condition-specific transformation of the scalp fields. The choice of baseline procedure should therefore be determined by the experimental design, the comparison conditions, and the scientific question.

## 10 Conclusion and future directions

EEG microstates provide sensitive, sub-second, whole-brain indices of the developing brain’s functional dynamics (Koenig et al., 2002). This tutorial demonstrates how microstate analyses integrated within HAPPE can provide reproducible, transparent results tailored for pediatric EEG. Embedding microstate computation into standardized pipelines not only minimizes subjective analytic decisions but also facilitates cross-cohort harmonization and meta-analysis, maximizing the interpretive power of microstate analyses across large pediatric cohorts. EEG microstate analysis offers unique advantages for developmental populations, offering temporally precise network-level metrics from brief, naturalistic recordings by quantifying rapid state switching in the presence as well as absence of controlled task conditions. Our task microstate approach should be interpreted as reflecting broad task-related topographic states rather than polarity-specific time-locked components. It provides large-scale network-level empirical markers that could be complemented with spectral and EEG connectivity measures like coherence and phase-lag indices to deduce brain functions. We encourage the field to adopt transparent, age-appropriate parameterization measures (e.g., data-driven class solutions) and open benchmarks (shared codes and test datasets) to evaluate robustness across analysis choices for microstate computation in developmental datasets. We hope that in these ways this tutorial facilitates future research examining the dynamics of brain network development with EEG tools.

## Data Availability

The datasets presented in this study can be found in online repositories. The names of the repository/repositories and accession number(s) can be found at: https://zenodo.org/records/17527065.

## Footnotes

1
https://zenodo.org/records/17527065

2
https://github.com/PINE-Lab/HAPPE/blob/master/3.%20generate/generateMicroStates.m
